# Supplementary material for: Iterative SCRaMbLE for engineering synthetic genome modules and chromosomes
Source: Nat Commun. 2025 Aug 7;16:7278. doi: 10.1038/s41467-025-62356-y (PMC12331891; doi:10.1038/s41467-025-62356-y)
Supplement: Supplementary file 1 — Supplementary Information [file 41467_2025_62356_MOESM1_ESM.pdf]

Supplementary material for the publication:

Iterative SCRaMbLE for Engineering Synthetic Genome Modules and Chromosomes

Xinyu Lu[1,2], Klaudia Ciurkot[1,2], Glen-Oliver F. Gowers[1,2], William M Shaw[1,2,3] and Tom Ellis[1,2*]

1. Imperial College Centre for Synthetic Biology, Imperial College London, London, UK
2. Department of Bioengineering, Imperial College London, London, UK
3. Department of Biomedical Engineering, Boston University, Boston, MA 02215, USA

* Correspondence should be sent to: t.ellis@imperial.ac.uk

Supplementary Figures

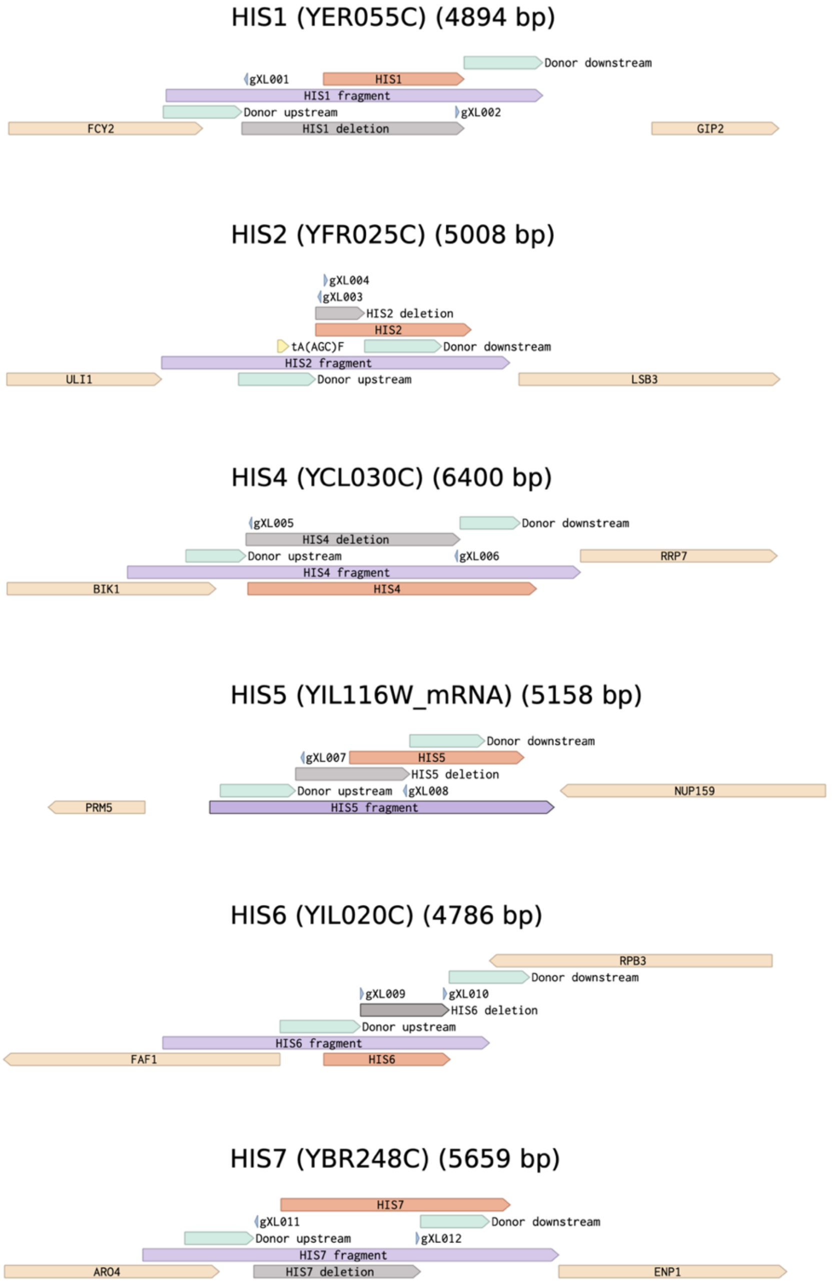

Figure S1. Schematic for gene deletion and defragmentation of *HIS* genes on Benchling (www.benchling.com). The CDS of genes are indicated by dark orange. Neighbouring genes are marked in light orange. Deletion regions are highlighted in grey. Donor parts for marker free gene deletion, each containing 500 bp homologous sequence that integrates into the target gene locus via homologous recombination, are shown in green. Gene fragments for defragmentation of the synthetic *HIS* module are shown in purple. tRNA gene tA(AGC)F upstream of *HIS2* is marked in yellow.

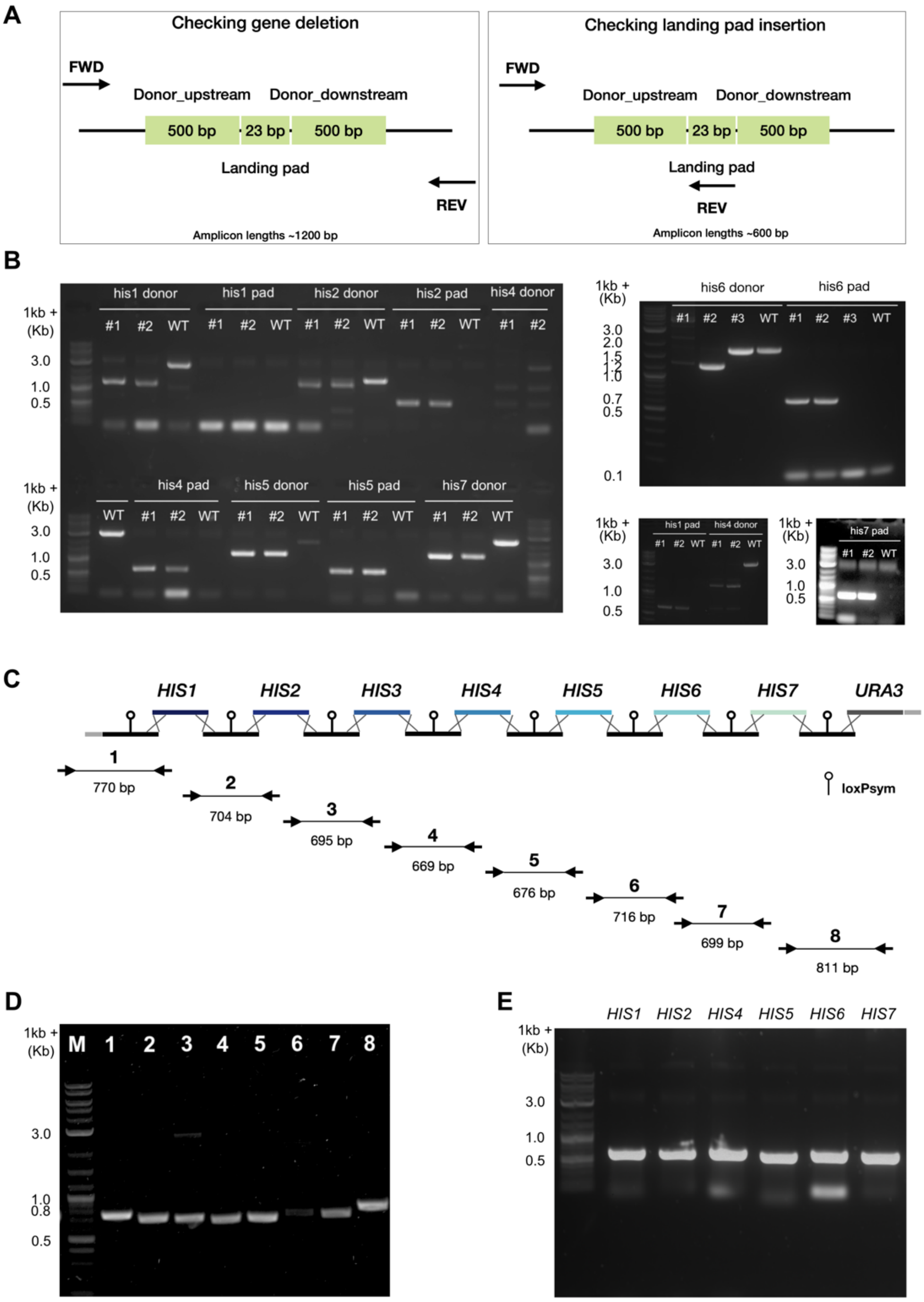

Figure S2. Junction PCR to verify gene deletions and synthetic module assembly. (A) Schematic of primer design to identify *HIS* gene deletion and landing pad insertion. (B) Colony PCR of transformants to identify *HIS* gene deletion. Numbers in white colour indicate the various tested colonies. (C) Schematic of synthetic *HIS* module assembly by yeast homology-dependent recombination and primer design to identify synthetic *HIS* module integration. Arrows in black represent the primers targeting at the junctions for PCR. Lines connected the arrows represent the various tested junctions, with numbers above indicating each junction and expected amplicon lengths shown below the lines. (D) Junction PCR from the genomic DNA of strain yXL052 for confirming the defragmented *HIS* cluster integration. (E) PCR from genomic DNA of strain yXL052 for confirming the landing pad insertion at the *HIS* gene deletion sites.

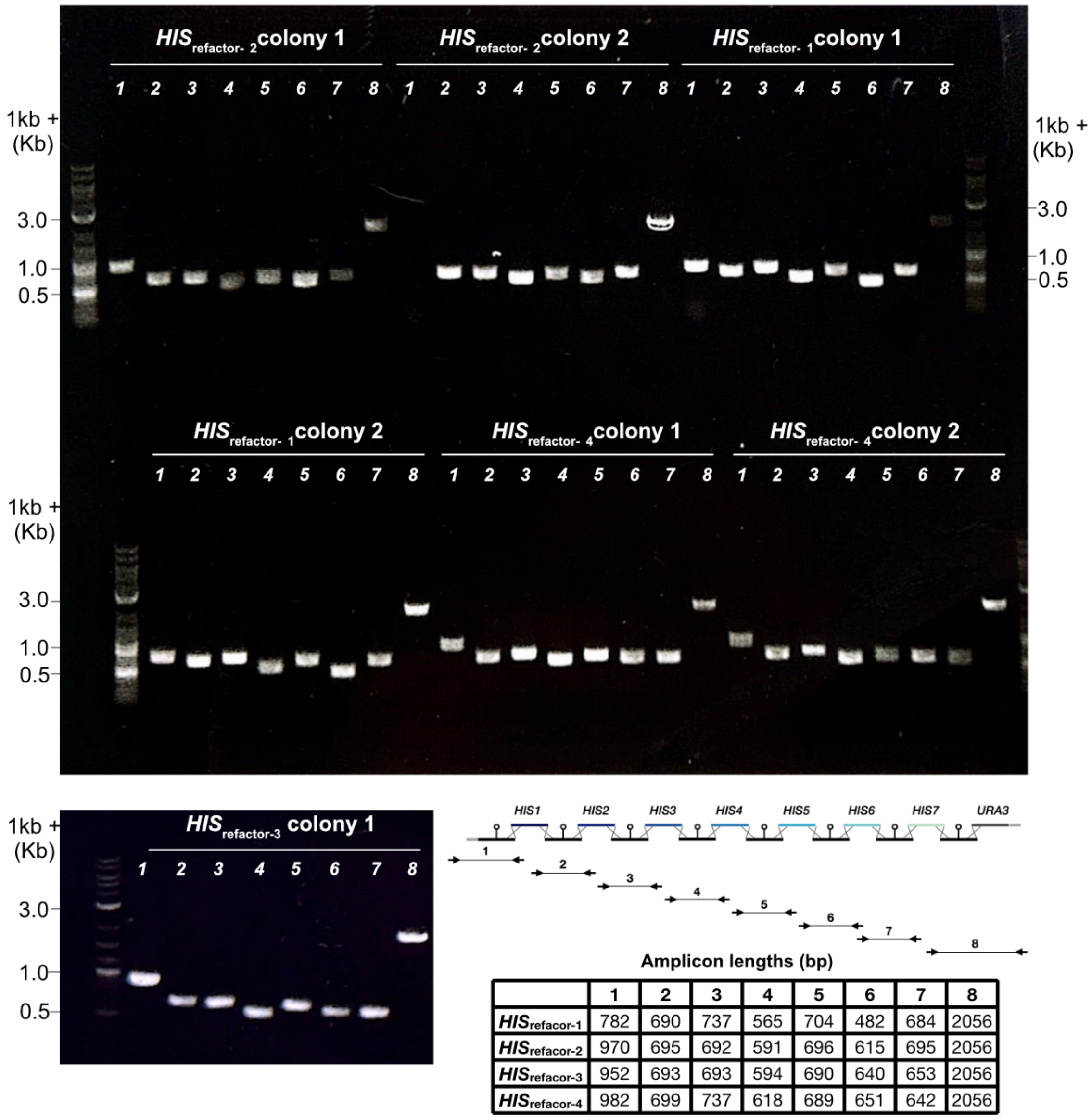

Amplicon lengths (bp)

	1	2	3	4	5	6	7	8
HISrefacor-1	782	690	737	565	704	482	684	2056
HISrefacor-2	970	695	692	591	696	615	695	2056
HISrefacor-3	952	693	693	594	690	640	653	2056
HISrefacor-4	982	699	737	618	689	651	642	2056

Figure S3. Junction PCR to verify the assembly of the refactored *HIS* modules. Transformants were randomly selected for checking the synthetic module assemblies. Eight junctions were checked for each strain. Arrows in black represent the primers targeting at the junctions for PCR. Lines connecting the arrows represent the various tested junctions, with numbers above indicating each junction. Expected amplicon lengths are listed in the table.

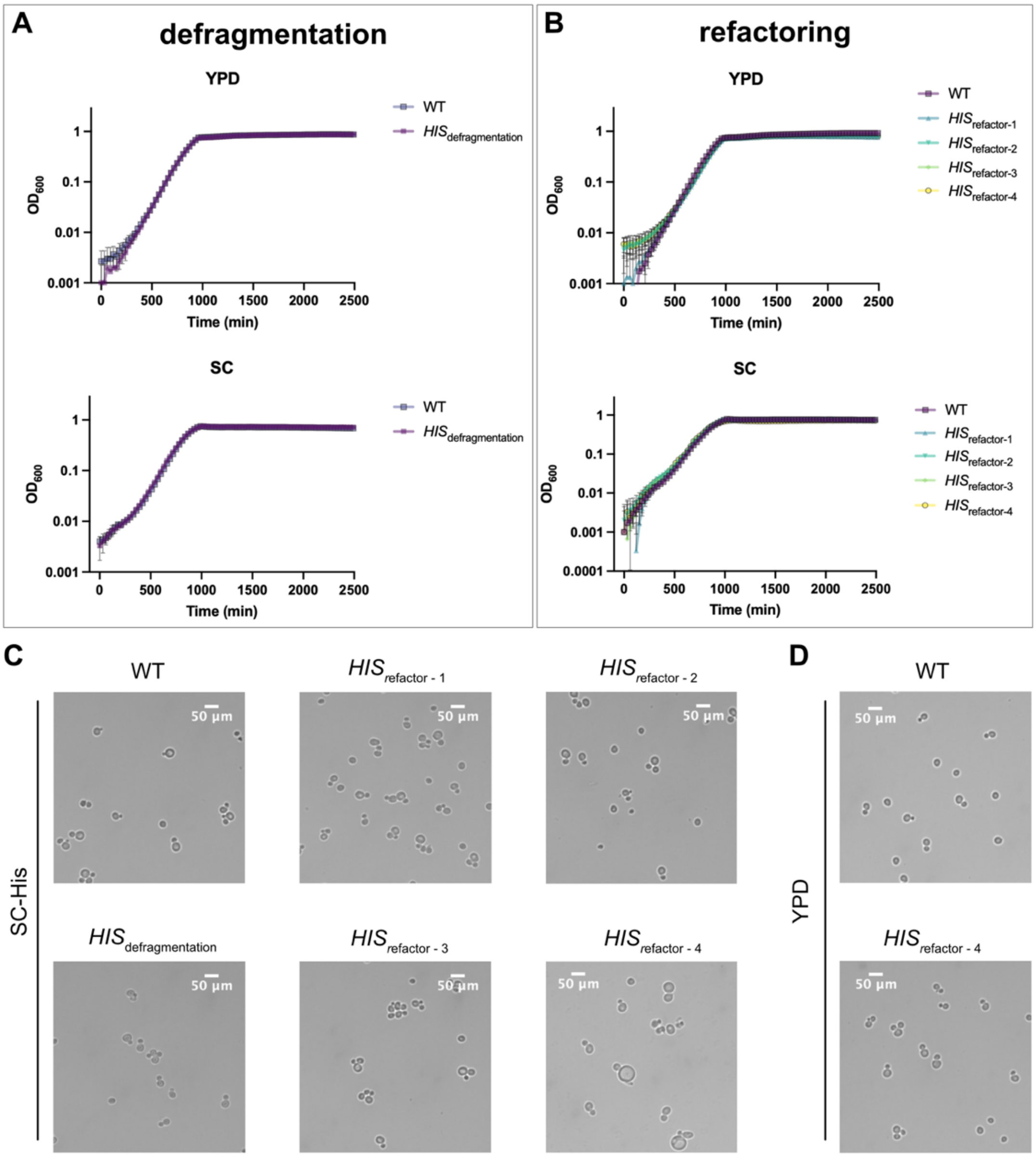

Figure S4. Physiological characterisation of the strains harbouring the defragmented and refactored *HIS* cluster. (A) Growth curves of the WT control strain (blue, yXL014) and the strain harbouring the defragmented *HIS* module (purple, yXL052) in YPD and SC media. Mean OD$_{600}$ from 3 biological replicates are shown as squares, with error bars representing standard deviation. (B) Growth curves of the WT control strain (yXL014, purple, $n = 4$ biologically independent samples) and the strains harbouring the refactored *HIS* modules (yXL214, yXL215, yXL269 and yXL216), in which *HIS* genes are driven by high (*HIS*$_{\text{refactor-1}}$, light blue), medium (*HIS*$_{\text{refactor-2}}$, cyan), low (*HIS*$_{\text{refactor-3}}$, light green) and mixed strength (*HIS*$_{\text{refactor-4}}$, yellow) promoters respectively, in YPD and SC media ($n = 3$ biologically independent samples). Mean values are plotted and error bars indicate standard deviation. (C) Microscopy images of the WT control strain (yXL014) and strains harbouring the synthetic *HIS* modules from the overnight culture in SC-His. (D) Microscopy images of the WT control strain (yXL014) and the strain harbouring the refactored *HIS* cluster (*HIS*$_{\text{refactor-4}}$) from the overnight culture in YPD. Source data are provided as a Source Data file.

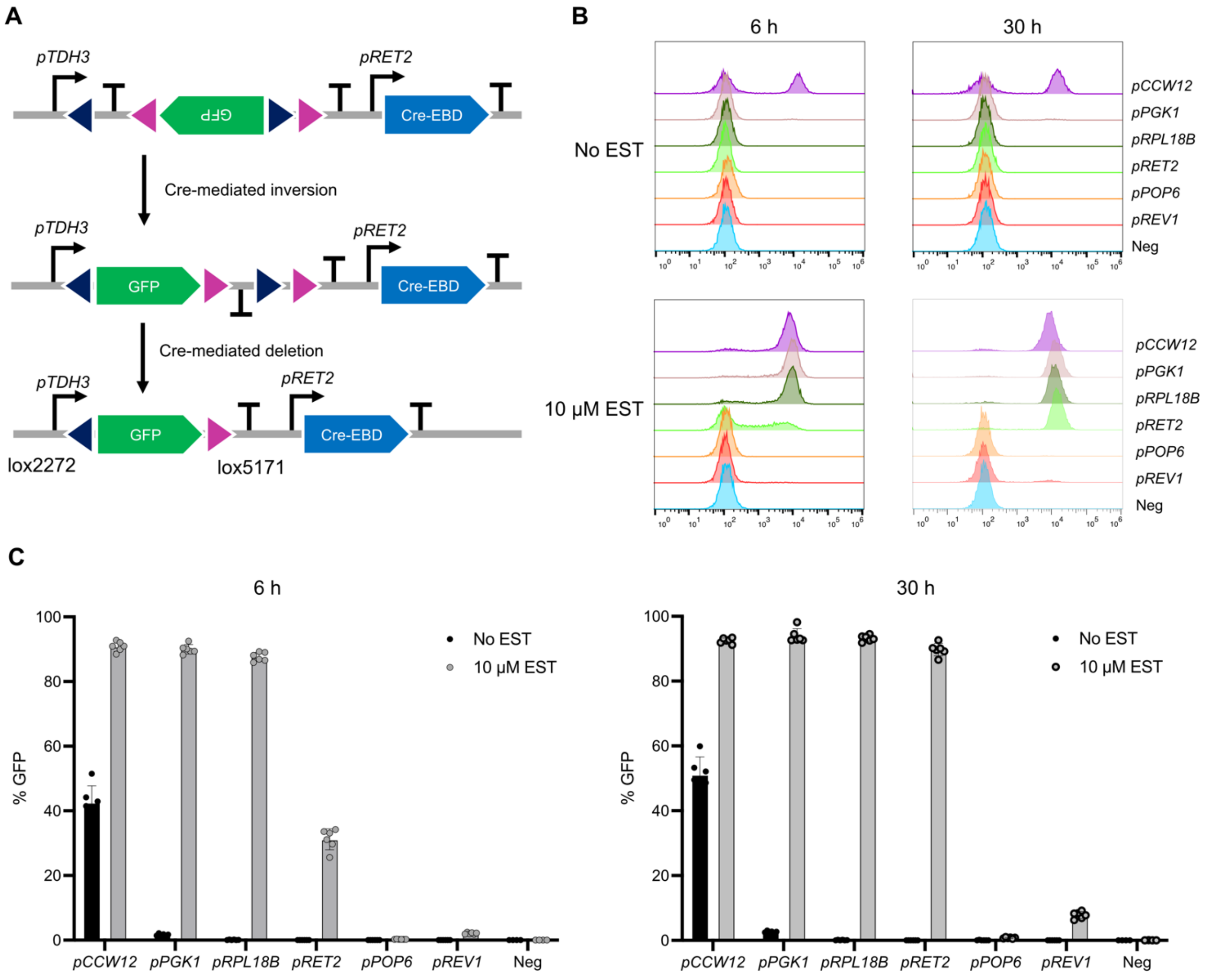

Figure S5. Characterisation of promoters for inducible Cre-EBD expression. (A) SCRaMbLE reporter constructs integrated at the *URA3* locus for characterisation of Cre-EBD expression promoters. RET2 promoter was finally selected as it showed zero leak and the cells fully convert to GFP⁺ over time. (B) Histograms of GFP fluorescence measured by flow cytometry after back dilution of saturated overnight cultures into fresh SC medium with and without 10 μM β-estradiol (EST) for 6 h (left) and 30 hours (right). (C) Percentage of the GFP⁺ cells in a yeast population measured by flow cytometry after back diluting saturated overnight cultures ($n = 6$ biologically independent samples) into fresh SC medium with (black) and without (grey) 10 μM β-estradiol (EST) for 6 h (left) and 30 hours (right). Percentages were calculated from flow cytometry data shown in panel B, using a gating strategy that select single yeast cells and define GFP⁺ and GFP⁻ populations based on a negative control ($n = 4$ biologically independent samples). Source data are provided as a Source Data file.

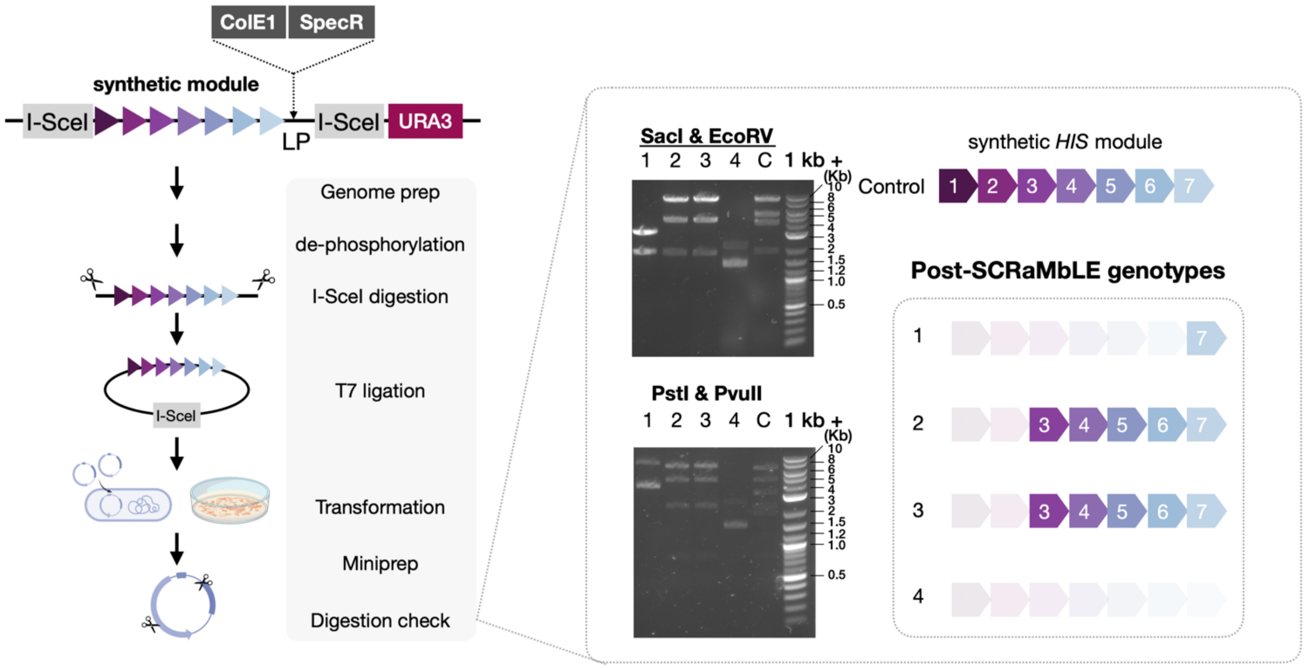

Figure S6. Analysis of the post-SCRaMbLE rearrangements using restriction digestion. Left: The ColE1 origin of replication and a selectable marker (*SpecR*) is inserted at the site of the landing pad (LP) between the synthetic module and the downstream I-SceI restriction site. The genomic region flanking by two I-SceI sites is then digested and ligated into a circular DNA through I-SceI digestion and T7 ligation. The ligation product is transformed into *E.coli* to enrich the circular plasmid containing the synthetic module. After miniprep, various combinations of restriction digestion are performed to identify the gene rearrangement patterns post SCRaMbLE. Right: Digestion results and schematic showing the corresponding genotypes from 4 post-SCRaMbLE strains and a control strain from the uninduced sample, labelling as "1, 2, 3, 4 and C" in black, respectively. Numbers in white represent 7 *HIS* genes (*HIS1* to *HIS7*). Arrows indicate the direction of gene transcription. Some icons for illustrating the workflow are created with BioGDP.com[1].

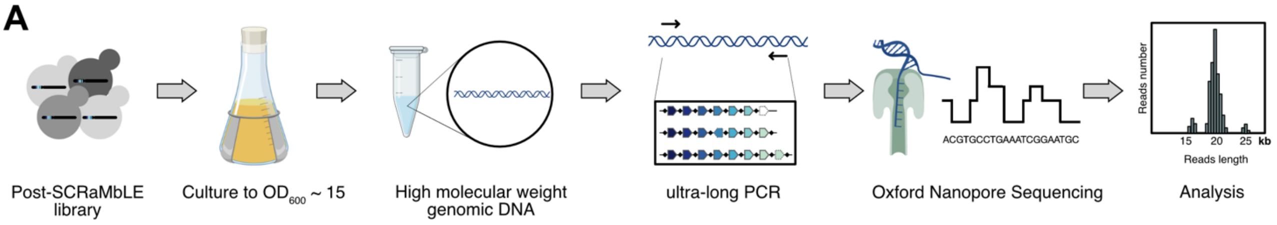

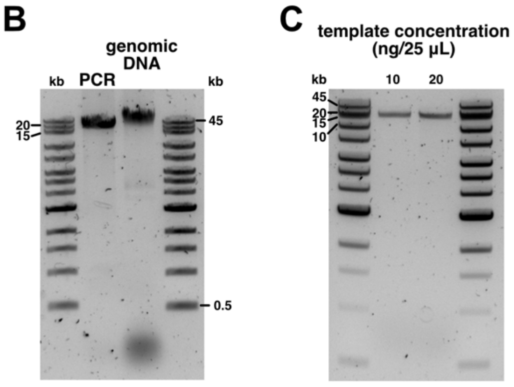

D

	yXL219$_{pool}$	yXL327$_{pool}$	yXL397$_{pool}$
**Round of SCRaMbLE**	1	2	2
**Parental strain**	yXL219 (*HIS*$_{refactor-4}$)	yXL219$_{SCRaMbLE1-2}$	yXL219$_{SCRaMbLE1-13}$
**Kit and Flowcell**	LSK109/FLG001	LSK114/FLG014	LSK114/FLG014
**N50**	19.98 kb	17.71 kb	19.44 kb
**Reads generated**	56,280	116,290	120,430
**Reads passed**	36,986	96,485	33,395
**Reads demultiplexed (FWD+ REV)**	6,635	5,104	6,853
**Reads annotated**	4,050	3,981	3,787
**Genotypes (All reads)**	127	353	261
**Genotypes (>10 reads)**	22	39	20

Figure S7. Workflow for POLAR-seq to identify gene rearrangements from the post-SCRaMbLE cell library. (A) The post-SCRaMbLE yeast cell library sorted through FACS are grown overnight to saturation (OD_{600} ~15) in appropriate media in a baffled flask. 50 mL cell culture are collected to isolate whole genomic DNA with high molecular weight (HMW), which is next used as the template in long PCR to obtain amplicons of the rearranged synthetic module for nanopore sequencing. Long reads of the amplicons are detected by identifying the primer sequences and further annotated by a custom python script[2]. Some icons for illustrating the workflow are created with BioGDP.com[1]. (B) Gel image showing the isolated HMW DNA of the post-SCRaMbLE library (yXL397$_{pool}$) and amplification of the rearranged synthetic *HIS* module using this isolated HMW DNA as the template. (C) Optimisation of template concentration for ultra-long PCR. 6 µL of each PCR product was checked by electrophoresis. (D) Statistics of nanopore sequencing runs of sample yXL219$_{pool}$, yXL327$_{pool}$ and yXL397$_{pool}$, each derived from distinct rounds of SCRaMbLE. The parental strains used in each SCRaMbLE round are listed in the table.

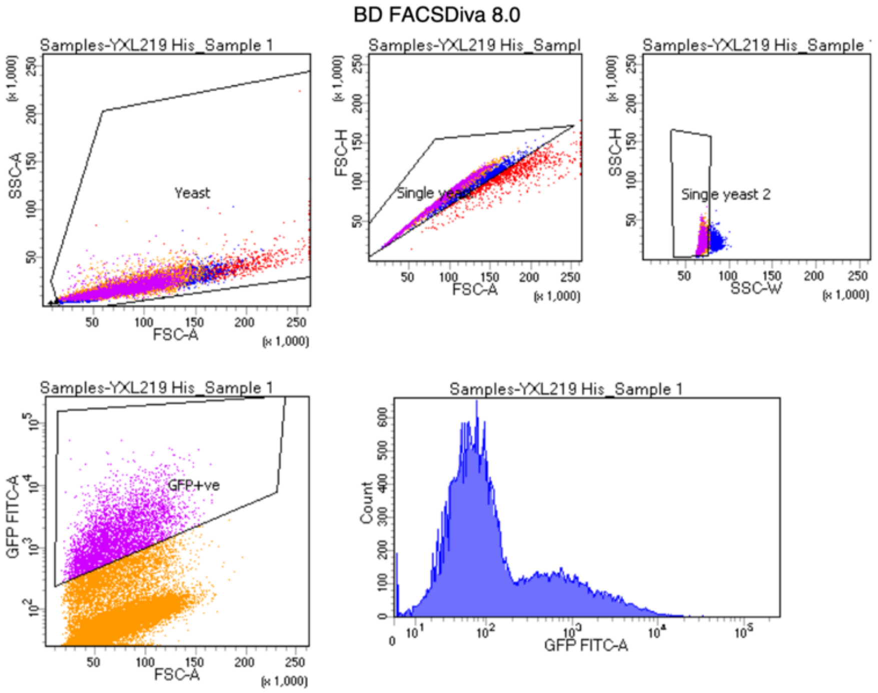

Tube: YXL219 His_Sample 1			
Population	#Events	%Parent	%Total
All Events	30,000	####	100.0
Yeast	29,917	99.7	99.7
Single yeast	28,912	96.6	96.4
Single yeast 2	27,270	94.3	90.9
GFP+ve	4,638	17.0	15.5

Figure S8. FACS sorting of post-SCRaMbLE cells using SCOUT. Strain yXL219 was SCRaMbLEd in SC-His medium for 4 hours. Cells were washed with water twice and resuspended in PBS buffer. Yeast cells were initially gated based on morphology (FSC-A vs SSC-A), followed by selection of single cells through doublet discrimination (FSC-A vs FSC-H and SSC-W vs SSC-H). GFP$^+$ single cells were subsequently identified based on fluorescence using the 530/30 bandpass filter (FSC-A vs GFP). Percentage of these gated populations is shown in the table at the bottom.

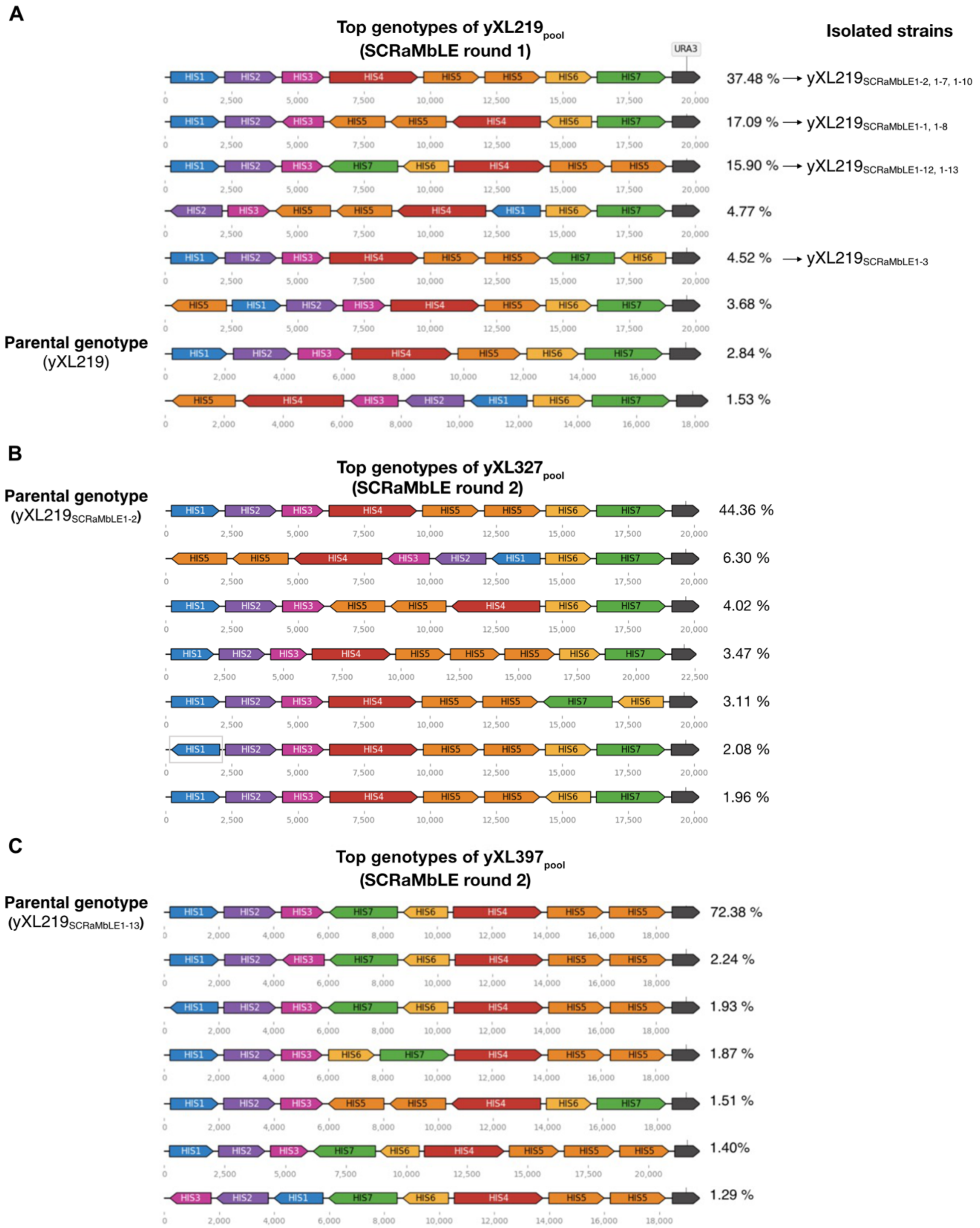

Figure S9. The most abundant genotypes identified from post-SCRaMbLE libraries across different SCRaMbLE rounds (A) yXL219$_{pool}$, (B) yXL327$_{pool}$ and (C) yXL397$_{pool}$. Transcription units (TUs) of the genes are illustrated in different colours, with their transcription direction indicated by arrows. Reads frequency of each genotype is shown on the right. Numbers in grey below each genotype annotate the positions (in bp) of each TU within the synthetic module. Strains isolated from yXL219$_{pool}$ with their genotypes identified by nanopore sequencing are indicated by arrows on the right. Parental strains used in each SCRaMbLE round are indicated on the left. Source data are provided as a Source Data file.

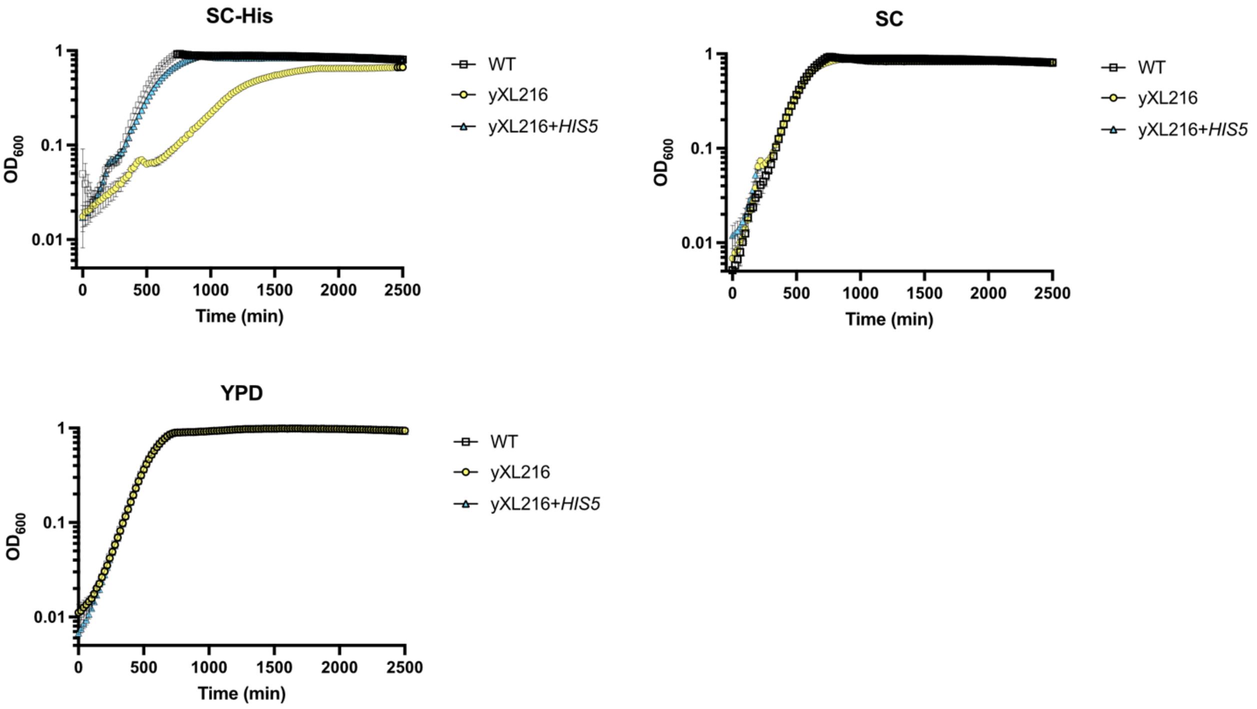

Figure S10. Growth restoration of the *HIS*$_{\text{refactor-4}}$ strain (yXL216) after integrating an additional copy of *HIS5* gene driven by the *RAD27* promoter at the *LEU2* locus. Growth curves of the WT control strain (yXL014+*LEU2*), the strain harbouring the refactored *HIS*$_{\text{refactor-4}}$ module (yXL216) and the strain yXL216 with an additional copy of the *HIS5* gene (yXL216+*HIS5*) driven by the *RAD27* promoter integrated at the *LEU2* locus. Growth was measured in SC-His, SC, and YPD media, n=3. Error bars represent standard deviation. Source data are provided as a Source Data file.

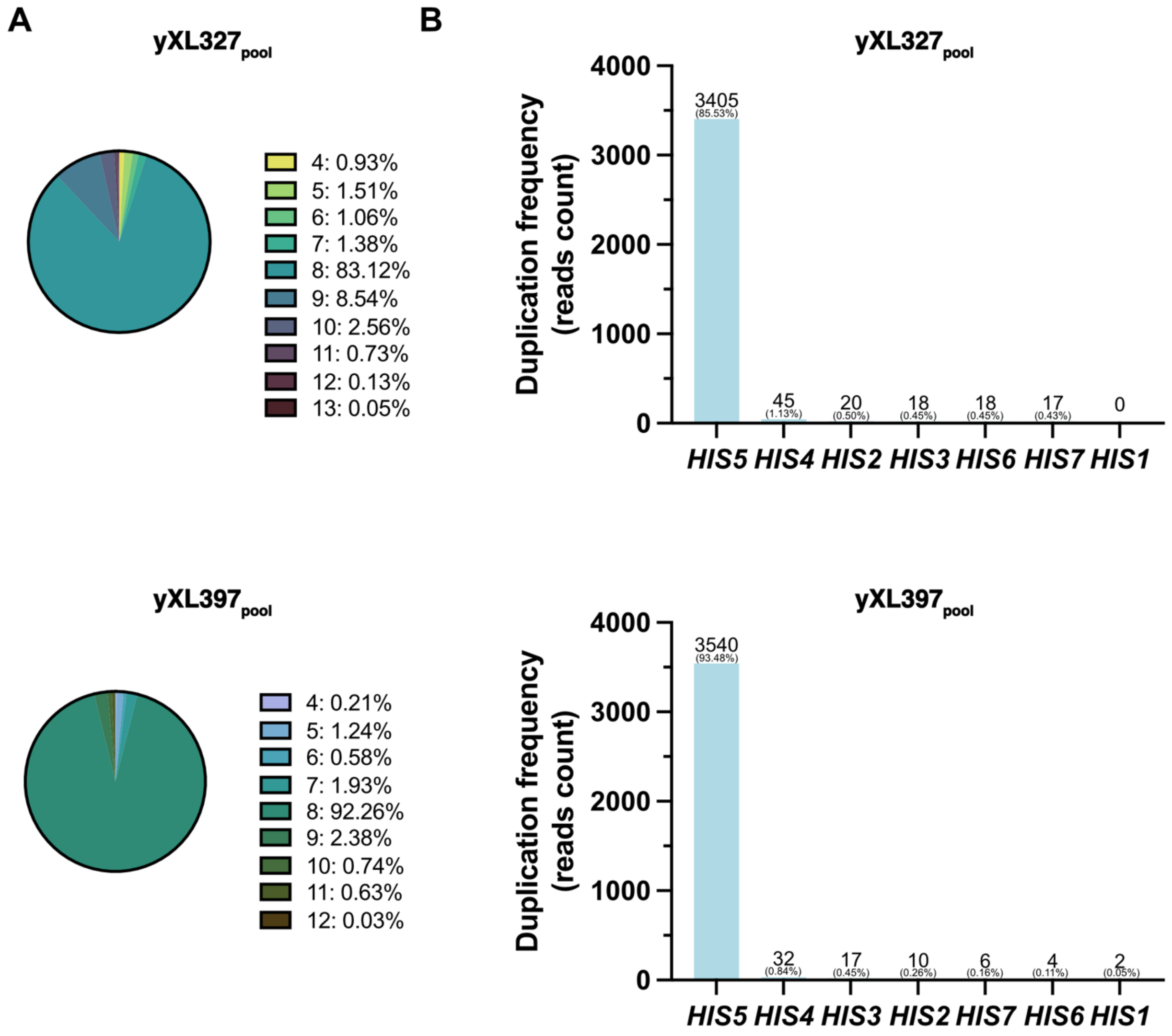

Figure S11. Genotypic composition of the post-SCRaMbLE libraries in the second round of SCRaMbLE. (A) Pie charts showing the number of genes identified from each distinct genotype and the percentage of total read count for each genotype featured with specific gene numbers. (B) Bar charts displaying the duplication frequency of each gene in the detected genotypes, calculated by the sum of reads count for genotypes detected with the duplicated genes dividing by the total read count in respective datasets. Source data are provided as a Source Data file.

Supplementary Tables

Supplementary Table 1. List of strains generated in this study.

Name	Genotype	Description	Parental	Source
**BY4741**	*MATa his3Δ1 leu2Δ0 met15Δ0 ura3Δ0*	Parental		[3]
**synV (yXZX846) -pBAB016**	*MATa his3Δ1 leu2Δ0 lys2Δ0 ura3Δ0 synV pBAB016* (*LEU2*)-*chrV_tRNA* (*HIS3*)	full replacement of native chrV with a synthetic chromosome V and transformed with a constitutive sfGFP expression plasmid pBAB016		[4]
**yXL014**	*MATa his3Δ1 leu2Δ0 met15Δ0 ura3Δ0 HIS3 URA3*	Control, BY4741 with *URA3* and *HIS3* prototroph	BY4741	this study
**yXL010**	*MATa his3Δ1 leu2Δ0 met15Δ0 ura3Δ0 his1Δ his2Δ his4Δ his5Δ his6Δ his7Δ*	BY4741 with *HIS1 HIS2 HIS4 HIS5 HIS6 HIS7* deletion (markerless)	BY4741	this study
**yXL052**	*MATa his3Δ1 leu2Δ0 met15Δ0 ura3Δ0 his1Δ his2Δ his4Δ his5Δ his6Δ his7Δ HIS1 HIS2 HIS3 HIS4 HIS5 HIS6 HIS7 at URA3* (*URA3*)	BY4741 with *HIS* defragmentation at *URA3* locus	yXL010	this study
**yXL214**	*MATa his3Δ1 leu2Δ0 met15Δ0 ura3Δ0 his1Δ his2Δ his4Δ his5Δ his6Δ his7Δ pCCW12-HIS1-tENO1 pHHF2-HIS2-tSSA1 pTDH3-HIS3-tADH1 pTEF1-HIS4-tPGK1 pHHF1-HIS5-tENO2 pTEF2-HIS6-tTDH1 pPGK1-HIS7-tTEF1 at URA3* (*URA3*)	yXL010 with refactored *HIS* cluster (*HIS*$_{\text{refactor-1}}$) integration at *URA3* locus, using strong promoters	yXL010	this study
**yXL215**	*MATa his3Δ1 leu2Δ0 met15Δ0 ura3Δ0 his1Δ his2Δ his4Δ his5Δ his6Δ his7Δ pHTB2-HIS1-tENO1 pALD6-HIS2-tSSA1 pHHF1-HIS3-tADH1 pPAB1-HIS4-tPGK1 pRNR1-HIS5-tENO2 pRET2-HIS6-tTDH1 pRPL18b-HIS7-tTEF1 at URA3* (*URA3*)	yXL010 with refactored *HIS* cluster (*HIS*$_{\text{refactor-2}}$) integration at *URA3* locus, using medium promoters	yXL010	this study
**yXL269**	*MATa his3Δ1 leu2Δ0 met15Δ0 ura3Δ0 his1Δ his2Δ his4Δ his5Δ his6Δ his7Δ pRET2-HIS1-tENO1 pRNR2-HIS2-tSSA1 pRPL18b-HIS3-tADH1 pPOP6-HIS4-tPGK1 pREV1-HIS5-tENO2 pRAD27-HIS6-tTDH1 pSAC6-HIS7-tTEF1 at URA3* (*URA3*)	yXL010 with refactored *HIS* cluster (*HIS*$_{\text{refactor-3}}$) integration at *URA3* locus, using weak promoters	yXL010	this study
**yXL216**	*MATa his3Δ1 leu2Δ0 met15Δ0 ura3Δ0 his1Δ his2Δ his4Δ his5Δ his6Δ his7Δ pPGK1-HIS1-tENO1 pHHF1-HIS2-tSSA1 pTDH3-HIS3-tADH1 pRPL18b-HIS4-tPGK1 pRAD27-HIS5-tENO2 pALD6-HIS6-tTDH1 pTEF1-HIS7-tTEF1 at URA3* (*URA3*)	yXL010 with refactored *HIS* cluster (*HIS*$_{\text{refactor-4}}$) integration at *URA3* locus, using mixed promoters	yXL010	this study
**yXL219**	*MATa his3Δ1 leu2Δ0 met15Δ0 ura3Δ0 his1Δ his2Δ his4Δ his5Δ his6Δ his7Δ pPGK1-HIS1-tENO1 pHHF1-HIS2-tSSA1 pTDH3-HIS3-tADH1 pRPL18b-HIS4-tPGK1 pRAD27-HIS5-tENO2 pALD6-HIS6-tTDH1 pTEF1-HIS7-tTEF1* at *URA3* (*URA3*) pXL005 (*LEU2*)	yXL216 transformed with SCRaMbLE reporter (pXL005)	yXL216	this study

Supplementary Table 2. List of gap repair donor and gene fragment plasmids used for defragmentation of *HIS* biosynthesis in this study.

To ease future engineering of the sites left behind by gene deletion, we substituted each deleted sequences with an individual 23 bp 'landing pad'[5] that encodes a unique CRISPR/Cas9 target sequence. Landing pad sequences are shown in the right column with protospacer adjacent motif (PAM) sequence highlighted by underlining.

Name	Insert description	Plasmid backbone	*E.coli* marker	Landing pad sequence[5]
pXL044	*HIS1* deletion donor	pYTK001	**CamR**	AATGCAATCGTAGTCCACCTCGG
pXL045	*HIS2* deletion donor	pYTK001	**CamR**	GATCGTACTTAGAAATGAGGCGG
pXL046	*HIS4* deletion donor	pYTK001	**CamR**	AATGGGGTTAGCAAGTCGCACGG
pXL047	*HIS5* deletion donor	pYTK001	**CamR**	CTAGCTTTCGTGTTAGTACGCGG
pXL048	*HIS6* deletion donor	pYTK001	**CamR**	TAGCATGGTGACACAAGCAGCGG
pXL049	*HIS7* deletion donor	pYTK001	**CamR**	CATCGCTTCCTACTTCCGCTCGG
pXL056	*HIS1* gene fragment	pYTK001	**CamR**	-
pXL057	*HIS2* gene fragment	pYTK001	**CamR**	-
pXL058	*HIS3* gene fragment	pYTK001	**CamR**	-
pXL059	*HIS4* gene fragment	pYTK001	**CamR**	-
pXL060	*HIS5* gene fragment	pYTK001	**CamR**	-
pXL061	*HIS6* gene fragment	pYTK001	**CamR**	-
pXL062	*HIS7* gene fragment	pYTK001	**CamR**	-

Supplementary Table 3. List of linker plasmids used in this study.

The loxPsym sequences are underlined.

Name	Insert	backbone	DNA sequence
pXL902	ConS with loxPsym	pYTK001	TGTCACACGAGGGAAACGACGGCAACCTACAACTTCTGGAGCCCGAAAAACAATAGAGACTTTACAGTGGGAC<br>CTATAACTTCGTATAATGTACATTATACGAAGTTATGCGTTCAATCGTATCGCCGCCTTCACGGGTTCTTATCTCAC<br>CGCTTTCTGTTGGAGTAGTTATTCCGAGTGAACG
pXL032	Con1 with loxPsym	pYTK001	GAACGAGCAGCAATACTACCAGACGCTACAACAGTTTATCAGGCACTTTCACTCCACGGTTCCTCTCTTCTTATTA<br>ATAACTTCGTATAATGTACATTATACGAAGTTATCTCAATCTCGGCAGGGCTACGGGTCGTCAGTAATCCAACCAC<br>CAGGCGGTCACCTCACTATCGTGTGCTTTATTG
pXL033	Con2 with loxPsym	pYTK001	AGTAAAAGTTATTGTCGGCTGTTGACTATTTCACGCAGTTCAGGGAAGCACCAGATTCTACGGACGGGATACTAC<br>ATAACTTCGTATAATGTACATTATACGAAGTTATTCGCTCCACCTCTCGGTAAAGGGTTCGCACGGCGTTCCTCAC<br>AGATACGATTTTCGGGTCTACAGTTGACGAGCA
pXL034	Con3 with loxPsym	pYTK001	CGACTGGTTTTCACTGTAAAGCGTGCCTGCGACGAAGAAGGGTAAGACGGCTCGGGACTGCCAATACGACAAC<br>CTATAACTTCGTATAATGTACATTATACGAAGTTATCCAGATAATAAGTTCAGCACCACGAAATAGTAGCAACGAGA<br>ATCGCCTCAGTAAGCGGGAAACACCGTAATACCT
pXL035	Con4 with loxPsym	pYTK001	TCGTGATAGGTAATCTGTCCTCAATAACACCGAAAGCGTCAGCAGGGAAGTGGCGGCTCTGGGTAAAAAGGTAG<br>TATAACTTCGTATAATGTACATTATACGAAGTTATAGGATTCAACGATAGCGTAATAAACTGTCAGGTGCTCTCTGC<br>GACCCTCAGCGAACGGGAGTGGAAAGTGTCTAC
pXL036	Con5 with loxPsym	pYTK001	CTTTATTCTACACAGACCCAGTGAGCCCAACAGTCGGCGTGGCAGTAAGCCTCTTCTCTATTTCCGTAGCACCTT<br>ATAACTTCGTATAATGTACATTATACGAAGTTATCAGTTTTCGCTGTAACCTCGCCCGCACTTTTATCACCAGGAGT<br>AGGCAACCACCCTTCGTTCAACTATTGTTCCA
pXL037	Con6 with loxPsym	pYTK001	GTTCCGACCTGCGGTAAACGAAAGTTATCTGCTCTGGCGTAAGTCTCCTACTGTGTCTGAATGCCCCTGATCAAT<br>ATAACTTCGTATAATGTACATTATACGAAGTTATGATGAGGCTATTGTAAAGCGAAGTTGGGAAGGTATCGGTCTAC<br>GGTAACGGAACCACTATCTCGGAGCACACGAC
pXL038	Con7 with loxPsym	pYTK001	TGAGGAACCTTTTACCCGCTGGGCTGACAGTATCTACGATTTATTGGCGAACACCCTGAAGAAACTTACGCACTA<br>ATAACTTCGTATAATGTACATTATACGAAGTTATTCCCACCGACAACTGACCACTCTATTACACGACCTCCGCTTAT<br>TTCCGTCTGGCTCGCTCTCACCCACAACAGTA
pXL039	Con8 with loxPsym	pYTK001	GCCGTATTCTATTTCGTCGGGAGAGTTCAGTCACAGGACCGTGTCTACAGATAAGGCAATCGTGCGTTGGGCAG<br>TATAACTTCGTATAATGTACATTATACGAAGTTATTTTTACTATTCCAGGGACTTCTTCGGCGAGAGGAGCAGGTCA<br>ATCCGTTTCGTATTACTGAGGTTGGTAGTGCCA
pXL040	Con9 with loxPsym	pYTK001	ACTGGAAGCACACGCTGTCCGCAGGTTGGAGCAATAGACTGATAATCTGGGATAGTAAGGGAGGCTGACTTTTC<br>GATAACTTCGTATAATGTACATTATACGAAGTTATGGTAAACTTCTGTTCGTAACTGTATTCGCCGTTCTCGTGGAT<br>TGTAGGAGTAGAGGACTGCTCGGTGATTCCGCA
pXL018	Con10 with loxPsym	pYTK001	CCATACCCTCCTAGTTCCCCGGTTATCTTTCCGAAGTGGGAGTGAGCGAACCTCCGTTTACGTCTTGCAATATAA<br>CTTCGTATAATGTACATTATACGAAGTTATGATGGATGTAGCTATGCACTTTGTACAGGGTGCCAACGGGTTTCAC<br>AATTCACAGATAGTGGGGATCCCGGCAAAGGGC
pXL019	Con11 with loxPsym	pYTK001	TGCTCGCCACTGCCGAAAGTTCGTACCGCTCATTCACTAGGTTGCGAAGCCTATGCTGATATATGAATCCACAAT<br>ATAACTTCGTATAATGTACATTATACGAAGTTATGATGCAGGGCTCTTAAGATTCGGAGTTGTACATATTCATAACTC<br>CAATCGGCTTTTACGTGCACCACCGCGGGCG
pXL041	Con13 with loxPsym	pYTK001	GGACCTCTGGTTCATCCCGTGGGATATCAAGCTTCGTCTTGATAAAGCCCACGCCTCGGGTGTAGCAGAGAATA<br>ACTTCGTATAATGTACATTATACGAAGTTATGGACGCCTACTGAATTGTGCGATCCCTGCACCTCAGCTAAGGTAG

			CTACCATATCTGAGTTTCTAAGCCTTGCGACAGA
pXL042	Con14 with loxPsym	pYTK001	TAGATTATGAGGTTAGCCGAAAATGCACGTGGTGCCACCCGCCGACTGCTCCCTGAGTGTGGCTCTTTGTTCTGTATAACTTCGTATAATGTACATTATACGAAGTTATCAACGCCCGACCTTCATCGCGGCCGATTCCTTCTGCGGACCATGTCGTCCTGATACTTTGGCCATGTTTCCGTTG
pXL043	Con16 with loxPsym	pYTK001	AGGATAAGGGTAAACATACAAGTCGATAGAAGATGGTAGGCCGGGTTCAATTCACAACACTCTACGGCTCCTCATAACTTCGTATAATGTACATTATACGAAGTTATCGAGAGCTAGTAGGGCACCCTGTAGTTGGAAGGGGAACTATTTCGTGGAGGTAGCCCATACCGTGTCTCTTGCGG

Supplementary Table 4. List of oligos used in this study.

Name	Sequence (5' to 3')	Description	Use for
XL575	GGGCGGATTACTACCGTT	*URA3* 5’ F	check *URA3* integration
XL576	GATTTGGTTAGATTAGATATGGTTTC	*URA3* 3’ R	check *URA3* integration
KC093	GTGGCTGTGGTTTCAGGGTCCA	POLAR-seq URA3 5’ F	Long-range PCR
KC094	GAAATCATTACGACCGAGATTCCCG	POLAR-seq URA3 3’ R	Long-range PCR
XL116	CAAGAGTAGTCTGGACAATG	*HIS1*_KO_F	verification of *HIS* gene deletion
XL117	GAGAAGCGTAGCTGATACAC	*HIS1*_KO_R	verification of *HIS* gene deletion
XL141	GGACTACGATTGCATTAATTAG	*HIS1*_LP_R	verification of *HIS* gene deletion
XL119	GAGGAAGAGGTGATAATA	*HIS2*_KO_F	verification of *HIS* gene deletion
XL120	CTCTGGTGATTGGCTTTC	*HIS2*_KO_R	verification of *HIS* gene deletion
XL121	CCTCATTTCTAAGTACGATC	*HIS2*_LP_R	verification of *HIS* gene deletion
XL143	GGAACAATTACGCAACATAG	*HIS4*_KO_F	verification of *HIS* gene deletion
XL142	CTACTGGAAATCCTTTGGG	*HIS4*_KO_R	verification of *HIS* gene deletion
XL124	CCGTGCGACTTGCTAAC	*HIS4*-LP-R	verification of *HIS* gene deletion
XL125	GCTGTCGCATTAAGAATATC	*HIS5*_KO_F	verification of *HIS* gene deletion
XL126	CCAATTGGTAATATAACTTC	*HIS5*_KO_R	verification of *HIS* gene deletion
XL127	GCGTACTAACACGAAAGC	*HIS5*-LP-R	verification of *HIS* gene deletion
XL128	GAACTGTCTCTGGTATCTACTTC	*HIS6*_KO_F	verification of *HIS* gene deletion
XL140	CATAGAGTTCGAGTATGATC	*HIS6*_KO_R	verification of *HIS* gene deletion
XL130	GCTTGTGTCACCATGCTAT	*HIS6*-LP-R	verification of *HIS* gene deletion
XL131	GAACACTGCTTCGTTATTC	*HIS7*_KO_F	verification of *HIS* gene deletion
XL132	GTAAATACTCCTTTACATCG	*HIS7*_KO_R	verification of *HIS* gene deletion
XL133	CGAGCGGAAGTAGGAAG	*HIS7*-LP-R	verification of *HIS* gene deletion
XL646	CCTTTTCGGTCGTTTTTC	pALD6_conj_REV	verification of *HIS* refactoring
XL647	GCCGTAATATCTCTCCTTGAC	pHHF1_conj_REV	verification of *HIS* refactoring
XL648	GGATGGGAGTTGGTCATTTAG	pHHF2_conj_REV	verification of *HIS* refactoring
XL649	CTTAGGCCAGAGATTACAACATG	pHTB2_conj_REV	verification of *HIS* refactoring
XL650	CTTTACGGTGCCCTATAGTACAC	pPAB1_conj_REV	verification of *HIS* refactoring
XL651	CAGGAAGTCGAGCGTGTC	pPGK1_conj_REV	verification of *HIS* refactoring
XL652	CAAGAGAGATGCAGTAAAAACTG	pPOP6_conj_REV	verification of *HIS* refactoring
XL653	GTGGCAAGCAAACTTATATCAC	pRAD27_conj_REV	verification of *HIS* refactoring
XL654	CAAACACTTTACTTGCCCTG	pRET2_conj_REV	verification of *HIS* refactoring
XL655	GGATCATAGCTTCTAATATATCTTCC	pREV1_conj_REV	verification of *HIS* refactoring
XL656	CTTTTTAAATTTGATTTGGGTG	pRNR1_conj_REV	verification of *HIS* refactoring
XL657	CGTGGGTGTGGTCTTTC	pRNR2_conj_REV	verification of *HIS* refactoring
XL658	CAGTGTGACATGGTTAGAATG	pRPL_conj_REV	verification of *HIS* refactoring
XL659	CCACGGTTTCTTCAGTCAC	pSAC6_conj_REV	verification of *HIS* refactoring
XL660	GAATGGACCTATGAACTGATGG	pTDH3_conj_REV	verification of *HIS* refactoring
XL661	CTGTTGCTTCCTATGTGATATG	pTEF1_conj_REV	verification of *HIS* refactoring
XL662	GGTTTTAAAACGAAAATTCTTATTC	tADH1_conj_FWD	verification of *HIS* refactoring
XL663	GCTTTTGATTAAGCCTTCTAGTC	tENO1_conj_FWD	verification of *HIS* refactoring

XL664	ATCATAGTTTAGAACACTTTATATTAACGA	tENO2_conj_FWD	verification of *HIS* refactoring
XL665	ATTGAATTGAATTGAAATCGATAG	tPGK1_conj_FWD	verification of *HIS* refactoring
XL666	GTGCGGCAATTGATAATAAC	tSSA1_conj_FWD	verification of *HIS* refactoring
XL667	AAGCAATCTTGATGAGGATAATG	tTDH1_conj_FWD	verification of *HIS* refactoring
XL668	TCATGCTCATGACATCTCATATAC	tTDH2_conj_FWD	verification of *HIS* refactoring
XL669	CTCGAGGGAGATTGATAAGAC	tTEF1_conj_FWD	verification of *HIS* refactoring
XL968	CATCTGGAATCTGAACTGC	pCCW12_conj_REV	verification of *HIS* refactoring
XL969	CCGCATGATTGATGTTATG	pTEF2_conj_REV	verification of *HIS* refactoring
XL190	CATCATCTCATGGATCTGC	*ura3*_homo_*HIS1*_conj_F	verification of *HIS* defragmentation
XL191	CGCCCTAATCATTACTTCC	*ura3*_homo_*HIS1*_conj_R	verification of *HIS* defragmentation
XL192	GAAACCTTCTTCTTCAACC	*HIS1*_*HIS2*_conj_F	verification of *HIS* defragmentation
XL193	GATAATTATCATTTACCTACGC	*HIS1*_*HIS2*_conj_R	verification of *HIS* defragmentation
XL194	CTGATTATCTTGAACTGGATG	*HIS2*_*HIS3*_conj_F	verification of *HIS* defragmentation
XL195	ACAGAGCTGGTGGACAAG	*HIS2*_*HIS3*_conj_R	verification of *HIS* defragmentation
XL196	CCTATGAATGTCAGTAAGTATG	*HIS3*_*HIS4*_conj_F	verification of *HIS* defragmentation
XL197	CAAGTAGCTGTTCATTCTC	*HIS3*_*HIS4*_conj_R	verification of *HIS* defragmentation
XL198	GAGGTAGTAGCAAGAGTGGG	*HIS4*_*HIS5*_conj_F	verification of *HIS* defragmentation
XL199	GCTATCTGATCGATTACTTGG	*HIS4*_*HIS5*_conj_R	verification of *HIS* defragmentation
XL200	CCCATGAGGAGAACACAC	*HIS5*_*HIS6*_conj_F	verification of *HIS* defragmentation
XL201	GCAGCAATTTCTGAGAGAG	*HIS5*_*HIS6*_conj_R	verification of *HIS* defragmentation
XL202	GGAATGAAAAGCAAGGTTAG	*HIS6*_*HIS7*_conj_F	verification of *HIS* defragmentation
XL203	CTTAGCGTCGTAGTTGGTAC	*HIS6*_*HIS7*_conj_R	verification of *HIS* defragmentation
XL204	GAGGATGTACAGCTTGGAG	*HIS7*_*ura3*_conj_F	verification of *HIS* defragmentation
XL205	CAACTAACTCCAGTAATTCCTTG	*HIS7*_*ura3*_conj_R	verification of *HIS* defragmentation

Supplementary Table 5. List of gRNAs used in this study.

Name	Target	Guide	Purpose
gXL001	*HIS1* 5'	TTATTGTAAGACTGATATGC	*HIS*1 deletion
gXL002	*HIS1* 3'	ACTCGGCGCATCTGATATCA	*HIS*1 deletion
gXL003	*HIS2* 5'	TATAGTCACCGGAGTGTGAA	*HIS*2 deletion
gXL004	sequence within *HIS2*	GGATTCCGTGGTCGATCAAG	*HIS*2 deletion
gXL005	*HIS4* 5'	TCATCAATTAACGGTAGAAT	*HIS*4 deletion
gXL006	*HIS4* 3'	ATTTTGAACATACATTTTGG	*HIS*4 deletion
gXL007	*HIS5* 5'	TGACTCCTAACAATTAACTT	*HIS*5 deletion
gXL008	*HIS5* 3'	GAGTACATAGAATATGTTGG	*HIS*5 deletion
gXL009	*HIS6* 5'	CCACAATTTCTACAAGTGGG	*HIS*6 deletion
gXL010	*HIS6* 3'	AGTTTGAAGACTGCTGTAGA	*HIS*6 deletion
gXL011	sequence upstream of *HIS7*	CATGATCACCTAATTAGCCG	*HIS*7 deletion
gXL012	sequence within *HIS7*	GACAGTCGGTGGGGGGATCA	*HIS*7 deletion
gXL038	*URA3* locus in BY4741	TCAGGGTCCATAAAGCTCCC	transient CRISPR plasmid for integration at the *URA3* locus

Supplementary Table 6. List of parts in YTK format used in this study.

Name	Part type	Part description	Source*	*E.coli* marker
pXL009	3	*HIS1*	PCR from BY4741 genome	**CamR**
pXL010	3	*HIS2*	PCR from BY4741 genome	**CamR**
pWS942	3	*HIS3*	Will Shaw	**CamR**
pXL011	3	*HIS4_G599A*	PCR from BY4741 genome	**CamR**
pXL012	3	*HIS5*	PCR from BY4741 genome	**CamR**
pXL013	3	*HIS6*	PCR from BY4741 genome	**CamR**
pXL014	3	*HIS7_A1341G*	PCR from BY4741 genome	**CamR**
pJCH021	2	*pSCW11*	Jack Ho	**CamR**
pJCH022	3	*cre*	Jack Ho	**CamR**
pTMP059	4	*tTEF1*	YTK addition	**CamR**

* Tom Ellis Lab members who provided the parts are acknowledged by name.

Supplementary Table 7. List of pre-assembled linker vectors used in this study.

Name	Linker type	2	3	4	Backbone*	*E. coli* Marker
pXL748	S/6	ConS with loxPsym	*gfp* dropout	Con6 with loxPsym	pWS042	**AmpR**
pXL749	6/9	Con6 with loxPsym	*gfp* dropout	Con9 with loxPsym	pWS042	**AmpR**
pXL750	9/11	Con9 with loxPsym	*gfp* dropout	Con11 with loxPsym	pWS042	**AmpR**
pXL751	11/13	Con11 with loxPsym	*gfp* dropout	Con13 with loxPsym	pWS042	**AmpR**
pXL324	13/14	Con13 with loxPsym	*gfp* dropout	Con14 with loxPsym	pWS042	**AmpR**
pXL543	14/16	Con14 with loxPsym	*gfp* dropout	Con16 with loxPsym	pWS042	**AmpR**
pXL544	16/10	Con16 with loxPsym	*gfp* dropout	Con10 with loxPsym	pWS042	**AmpR**

Supplementary Table 8. List of cassettes used in this study.

Name	Cassette type	2	3a-1	3a-2	3b	4	Vector	*E. coli* Marker	Yeast Marker	Yeast Vector
pXL001	1/E	pJCH021		pJCH022		pYTK051	pWS042	**AmpR**		
pXL003	S/1	pTDH3-lox2272-tCYC1-lox5171-mGFPmut2-lox2272-lox5171-tADH1					pWS041	**AmpR**		
pXL445	S/6	pYTK016		pXL009		pYTK051	pXL748	**AmpR**		
pXL446	6/9	pYTK018		pXL010		pYTK052	pXL749	**AmpR**		
pXL447	9/11	pYTK015		pWS942		pYTK053	pXL750	**AmpR**		
pXL448	11/13	pYTK019		pXL011		pYTK054	pXL751	**AmpR**		
pXL449	13/14	pYTK021		pXL012		pYTK055	pXL324	**AmpR**		
pXL558	14/16	pYTK020		pXL013		pYTK056	pXL543	**AmpR**		
pXL562	16/10	pYTK017		pXL014		pTMP059	pXL544	**AmpR**		
pXL450	S/6	pYTK010		pXL009		pYTK051	pXL748	**AmpR**		
pXL451	6/9	pYTK012		pXL010		pYTK052	pXL749	**AmpR**		
pXL452	9/11	pYTK009		pWS942		pYTK053	pXL750	**AmpR**		
pXL453	11/13	pYTK013		pXL011		pYTK054	pXL751	**AmpR**		
pXL454	13/14	pYTK015		pXL012		pYTK055	pXL324	**AmpR**		
pXL559	14/16	pYTK014		pXL013		pYTK056	pXL543	**AmpR**		
pXL563	16/10	pYTK011		pXL014		pTMP059	pXL544	**AmpR**		
pXL455	S/6	pYTK020		pXL009		pYTK051	pXL748	**AmpR**		
pXL456	6/9	pYTK023		pXL010		pYTK052	pXL749	**AmpR**		
pXL457	9/11	pYTK017		pWS942		pYTK053	pXL750	**AmpR**		
pXL458	11/13	pYTK024		pXL011		pYTK054	pXL751	**AmpR**		
pXL459	13/14	pYTK027		pXL012		pYTK055	pXL324	**AmpR**		
pXL560	14/16	pYTK025		pXL013		pYTK056	pXL543	**AmpR**		
pXL564	16/10	pYTK022		pXL014		pTMP059	pXL544	**AmpR**		
pXL460	S/6	pYTK011		pXL009		pYTK051	pXL748	**AmpR**		
pXL461	6/9	pYTK015		pXL010		pYTK052	pXL749	**AmpR**		
pXL462	9/11	pYTK009		pWS942		pYTK053	pXL750	**AmpR**		
pXL463	11/13	pYTK017		pXL011		pYTK054	pXL751	**AmpR**		
pXL464	13/14	pYTK025		pXL012		pYTK055	pXL324	**AmpR**		
pXL561	14/16	pYTK018		pXL013		pYTK056	pXL543	**AmpR**		
pXL565	16/10	pYTK013		pXL014		pTMP059	pXL544	**AmpR**		
pXL1047	-	pYTK025		pXL012		pYTK055	pWS064	**KanR**	**LEU2**	integration

Supplementary Table 9. List of multigene cassettes used in this study.

Name	Cassette type	1 (vector)	2	3	4	5	6	*E. coli* Marker	Yeast Marker	Yeast Vector
pXL004	o	pWS036	pXL003	pXL001				**KanR**	**LEU2**	low-copy
pXL005	o	pWS036	pXL003	pWS2409				**KanR**	**LEU2**	low-copy

Supplementary Table 10. Statistics on multiplexed nanopore sequencing (R1-5).

Raw reads

	Reads	Throughput (Mbp)	Mean read length (kb)	N50 (kb)	Genome coverage
**R1**	121,607	1,208	9.9	20.8	99.8
**R2**	121,791	1,133	9.3	18.6	93.6
**R3**	86,293	1,042	12.1	25.4	86.1
**R4**	131,687	1,370	10.4	21.0	113.2
**R5**	99,960	1,315	13.2	27.9	108.7

Canu-corrected reads

	Reads	Throughput (Mbp)	Mean read length (kb)	N50 (kb)	Genome coverage
**R1**	11,821	486	41.1	45.5	40.2
**R2**	12,976	485	37.4	41.0	40.1
**R3**	11,263	485	43.0	46.7	40.1
**R4**	11,770	490	41.6	48.1	40.5
**R5**	10,728	492	45.9	53.8	40.7

Supplementary References

1. Jiang, S. *et al.* Generic Diagramming Platform (GDP): a comprehensive database of high-quality biomedical graphics. *Nucleic Acids Research* **53**, D1670–D1676 (2025).
2. Ciurkot, K. *et al.* Combinatorial Design Testing in Genomes with POLAR-seq. 2024.06.06.597521 Preprint at https://doi.org/10.1101/2024.06.06.597521 (2024).
3. Baker Brachmann, C. *et al.* Designer deletion strains derived from Saccharomyces cerevisiae S288C: A useful set of strains and plasmids for PCR-mediated gene disruption and other applications. *Yeast* **14**, 115–132 (1998).
4. Blount, B. A. *et al.* Rapid host strain improvement by in vivo rearrangement of a synthetic yeast chromosome. *Nat Commun* **9**, 1932 (2018).
5. Shaw, W. M. *et al.* Engineering a Model Cell for Rational Tuning of GPCR Signaling. *Cell* **177**, 782-796.e27 (2019).
